# Pathogenesis of (smoking-related) non-communicable diseases—Evidence for a common underlying pathophysiological pattern

**DOI:** 10.3389/fphys.2022.1037750

**Published:** 2022-12-15

**Authors:** Wolfgang Kopp

**Affiliations:** Retired, Graz, Austria

**Keywords:** epigenome and exposome, comorbidities, oxidative stress, hyperinsulinemia and insulin resistance, subclinical inflammation, microbiome, psoriasis, multiple sclerosis

## Abstract

Non-communicable diseases, like diabetes, cardiovascular diseases, cancer, stroke, chronic obstructive pulmonary disease, osteoporosis, arthritis, Alzheimer’s disease and other more are a leading cause of death in almost all countries. Lifestyle factors, especially poor diet and tobacco consumption, are considered to be the most important influencing factors in the development of these diseases. The Western diet has been shown to cause a significant distortion of normal physiology, characterized by dysregulation of the sympathetic nervous system, renin-angiotensin aldosterone system, and immune system, as well as disruption of physiological insulin and oxidant/antioxidant homeostasis, all of which play critical roles in the development of these diseases. This paper addresses the question of whether the development of smoking-related non-communicable diseases follows the same pathophysiological pattern. The evidence presented shows that exposure to cigarette smoke and/or nicotine causes the same complex dysregulation of physiology as described above, it further shows that the factors involved are strongly interrelated, and that all of these factors play a key role in the development of a broad spectrum of smoking-related diseases. Since not all smokers develop one or more of these diseases, it is proposed that this disruption of normal physiological balance represents a kind of pathogenetic “basic toolkit” for the potential development of a range of non-communicable diseases, and that the decision of whether and what disease will develop in an individual is determined by other, individual factors (“determinants”), such as the genome, epigenome, exposome, microbiome, and others. The common pathophysiological pattern underlying these diseases may provide an explanation for the often poorly understood links between non-communicable diseases and disease comorbidities. The proposed pathophysiological process offers new insights into the development of non-communicable diseases and may influence the direction of future research in both prevention and therapy.

## 1 Introduction

Non-communicable disorders, like type 2 diabetes, cardiovascular diseases (CVD), cancer, stroke, chronic obstructive pulmonary disease (COPD), arthritis, mental health issues and other more are a major cause of death in almost every country. Lifestyle factors, especially poor diet and tobacco use, are considered to be the main influencing factors in the development of these diseases.

The tobacco epidemic is one of the major public health threats. Cigarette smoking is the most common form of tobacco use and a leading cause of preventable death worldwide. Despite numerous efforts to curb the spread of tobacco use, about six million people worldwide die each year as a result of tobacco smoking (Commar et al., 2015). According to WHO estimates, the global mortality rate from tobacco use (active and passive) is about 7.2 million per year (Lelieveld et al., 2020). Cigarette smoking has been linked to the development of CVD, COPD, hypertension, cancer, as well as many chronic systemic diseases with inflammatory components such as atherosclerosis, Crohn’s disease, rheumatoid arthritis, psoriasis, Graves’ ophthalmopathy and type 2 diabetes (Centers for Disease Control and Prevention, 2010; Hunter and Reddy, 2013). The mechanisms by which cigarette smoking induces and promotes these diseases are complex and interconnected and still not completely understood.

As outlined in a recent article by Kopp (2019), the Western diet causes a dysregulation of several important physiological factors that play a crucial role in the development of various non-communicable diseases like atherosclerosis, cancer, type 2 diabetes, neurodegenerative diseases, and many more. Development of oxidative stress (OxS), hyperinsulinemia and insulin resistance, subclinical inflammation, and dysregulation of the sympathetic nervous system (SNS), renin-angiotensin aldosterone system (RAAS), and immune system have been identified as major alterations in this context.

This paper addresses the question of whether cigarette smoking causes the same pathophysiological changes as described above and if the development of smoking-related diseases therefore follows the same pathogenetic pattern.

## 2 Oxidative stress, hyperinsulinemia and insulin resistance, subclinical inflammation, and dysregulation of the SNS, the RAAS and the immune system: Influence of tobacco smoking

### 2.1 Smoking and OxS

In the cell, mitochondrial respiration is one of the main sources of reactive oxygen species (ROS). ROS are also generated by non-mitochondrial sources such as the nicotinamide adenine dinucleotide phosphate oxidase (NOX) and NOX homologs, ROS-generating enzymes, and the β-oxidation of fatty acids in the peroxisome. In addition to ROS, organisms also generate reactive nitrogen species (RNS) (Shields et al., 2021). In the physiological state, the level of cellular ROS is stable in a dynamic equilibrium because eukaryotic cells have multiple antioxidant defense mechanisms. ROS and RNS play a dual role: on the one hand, they regulate biological and physiological processes as signaling molecules on the other hand, a disturbed balance between the oxidative and antioxidant systems of cells and tissues, resulting in excess ROS and RNS, can damage macromolecular targets such as lipids, proteins, and nucleic acids and disrupt redox signaling (Gough and Cotter, 2011; Forman and Zhang, 2021). OxS and nitrosative stress play an essential role in the pathogenesis of many chronic diseases, including, (but not limited to) atherosclerosis, COPD, type 2 diabetes, neurodegenerative diseases, and cancer (Forman and Zhang, 2021; Yoon et al., 2021).

Tobacco smoke contains a complex mixture of toxic chemicals in the particulate and gaseous phases, e.g., high concentrations of a host of ROS and RNS, like superoxide, nitric oxide (NO), and peroxynitrite. Exposure to cigarette smoke therefore leads to significant OxS (Caliri et al., 2021). In a human biomarker study, Jansen et al. (2014) investigated the influence of smoking on biomarkers of OxS, redox and antioxidant status in a healthy male population. The study confirmed that smokers have increased levels of OxS biomarkers and impaired antioxidant status. An animal study in Wistar rats demonstrated that relatively stable oxidants in the gaseous phase of cigarette smoke can pass through the pulmonary alveolar wall into the blood and increase systemic OxS (Yamaguchi et al., 2007). As shown in an *in vivo* human study by Padmavathi et al. (2018), smoking increased OxS by decreasing antioxidant status in both erythrocytes and platelets and higher nitrite/nitrate levels in erythrocytes. Using an *in vitro* mesencephalic cell model, Barr et al. (2007) showed that nicotine induces dose-dependent ROS concentrations that lead to activation of the stress-dependent NF-κB pathway in mesencephalic cells.

In addition to exogenous ROS, smoking causes mitochondrial dysfunction, which - in the form of a vicious circle - leads to an endogenous increase in ROS production and OxS (Miró et al., 1999; Boukhenouna et al., 2018). Furthermore, smoking contributes to an imbalance of the oxidant/antioxidant system, resulting in significantly lower levels of antioxidant enzymes (Al-Bashaireh et al., 2018; Boukhenouna et al., 2018). The number of cigarettes smoked daily has an impact on the degree of oxidative damage and the reduction of antioxidant defenses (Kamceva et al., 2016).

### 2.2 Smoking and insulin resistance

Insulin is a hormone that has a key function in cell metabolism. In healthy people, proper insulin secretion and the sensitivity of peripheral tissues to the effects of the hormone keep glucose levels within the normal range. Insulin resistance is manifested by impaired glucose uptake and oxidation, impaired ability to suppress lipid oxidation, and a decrease in glycogen synthesis. Hyperinsulinemia can be both a result and a driver of insulin resistance (Nolan and Prentki, 2019).

Numerous studies have demonstrated negative, apparently dose-dependent effects of cigarette smoking on peripheral insulin action (Facchini et al., 1992; Śliwińska-Mossoń and Milnerowicz, 2017). Smoking is associated with insulin resistance and hyperinsulinemia. Compared to non-smokers, chronic cigarette smokers are insulin resistant, hyperinsulinemic and dyslipidemic. Serum insulin concentrations have been shown to be higher in smokers than in non-smokers, even after adjusting for factors that influence insulin resistance (Facchini et al., 1992; Kim K.W. et al., 2017). A human clinical trial by Attvall et al. (1993), which examined the short-time effect of smoking on insulin sensitivity in a group of 7 healthy habitual smokers, showed that smoking acutely impairs insulin effects and leads to insulin resistance and compensatory hyperinsulinemia. The degree of insulin resistance was found to be related to the number of cigarettes/day (Eliasson et al., 1994). In another human clinical trial involving 20 chronic smokers and 20 age-, sex-, and BMI-matched healthy subjects, cigarette smoking was shown to acutely worsen glucose tolerance in both healthy non-smokers and habitual tobacco smokers after smoking as few as 3 cigarettes (Frati et al., 1996). Nicotine produced insulin resistance through ROS-induced downregulation of Nrf2 activity in cardiomyocytes *in vitro* (Li et al., 2019). In an animal study, ApoE gene knockout mice were exposed to electronic cigarettes, e-cigarettes without nicotine, or conventional cigarettes for 18 weeks. A control group received fresh air. The result of the study showed a significant decrease in insulin tolerance in the e-cigarette, e-cigarette without nicotine, and cigarette groups compared to the control group (Lan et al., 2020). In addition, chronic cigarette smoking appears to significantly worsen insulin resistance in patients with type 2 diabetes (Śliwińska-Mossoń and Milnerowicz, 2017). Eliasson et al. (1996) found in a clinical study that long-term use of nicotine gum was associated with insulin resistance and hyperinsulinemia, suggesting that nicotine is a component of cigarette smoke that leads to insulin resistance.

### 2.3 Smoking, SNS and RAAS

The RAAS and the SNS are major physiological systems which play an important role in normal physiology as well as in pathologic conditions. The SNS, one of the two divisions of the autonomic nervous system, regulates the function of virtually all human organ systems. The catecholamine neuroeffectors norepinephrine (NE) and epinephrine act as both neurotransmitters and circulating hormones. While NE is released primarily from the sympathetic nerve terminals, epinephrine is mainly secreted from the adrenal medulla (Benarroch, 2020).

Nicotine and particulate matter in tobacco smoke lead to a rapid increase in SNS activity. This is reflected in increased plasma catecholamine levels and is maintained by a positive feedback loop between SNS activity and ROS (Middlekauff et al., 2014; Xiao et al., 2019).

The RAAS plays a central role in the regulation of vascular tone and salt and fluid balance. In addition to the classical circulating RAAS with angiotensin II (ANG II) as the main effector, there is also a local or tissue RAAS that operates independently of the circulating RAAS mainly at the cellular level *via* local ANG peptide synthesis, and plays a role in tissue physiology and homeostasis. ANG II, produced from Ang I by an angiotensin-converting enzyme (ACE), binds to ANG II type 1 receptor (AT1R) and ANG II type 2 receptor. ACE homolog ACE 2 antagonizes the functions of ANG II by degrading ANG II to its biologically active product ANG (1-7), which acts *via* the Mas receptor (Santos et al., 2018; Nehme et al., 2019). In addition to the more direct effects of ANG II on the cardiovascular system, the RAAS promotes inflammation and tissue injury (Patel et al., 2017; Nehme et al., 2019). Nicotine and/or cigarette smoke have been reported to impair RAAS homeostasis in multiple organ systems by upregulating the ACE/ANG-II/AT1R axis and downregulating the compensatory ACE2/ANG-(1-7)/Mas receptor axis (Oakes et al., 2018). In an animal study on hamsters exposed to cigarette smoke, Wang et al. (2010) showed that cigarette smoke increases tissue ANG II levels, likely by increasing chymase activity. Similarely, a 6-month exposure of Sprague-Dawley rats to cigarette smoke significant increased ANG II levels and ACE protein expression and decreased ACE2 expression in lung tissue (Yuan et al., 2015). A prospective study on 108 smokers with non-diabetic chronic kidney disease treated with ACE inhibitor therapy showed that smoking attenuated the renal protection achieved by ACE inhibition (Roehm et al., 2017).

### 2.4 Smoking, immune system and inflammation

Subclinical inflammation is the result of release of pro-inflammatory cytokines from immune-related cells as part of a widespread activation of the innate immune system. When activated, cells of the innate immune system produce proinflammatory cytokines that coordinate local and systemic inflammatory responses as part of a defense mechanism designed to eliminate harmful stimuli and promote tissue repair. Chronic (subclinical) inflammation causes OxS, whereas the OxS response can stimulate the release of proinflammatory cytokines and trigger subclinical inflammation, suggesting that inflammation and OxS are pathophysiological events that are closely linked in a vicious cycle (Liguori et al., 2018; Valacchi et al., 2018). Several important functions of the immune system are mediated by toll-like receptors (TLR) and nuclear factor-κB (NF-κB). TLRs are important effectors of innate immunity, and their activation is one of the first defense mechanisms, leading to an immune response through the production of proinflammatory cytokines, type I interferons, and other inflammatory mediators. There is growing evidence that TLRs play an important role in promoting inflammation, OxS, and endothelial dysfunction (Hovland et al., 2015; Salvador et al., 2016; Lazaridis et al., 2021). Their expression is not limited to innate immune cells and immune tissues, but is found in all tissues, including cardiovascular, liver, pancreas, colon, small intestine, lung, kidney, ovary, placenta, testis, prostate, skeletal muscle, and brain (El-Zayat et al., 2019; Lazaridis et al., 2021). All TLR signaling pathways culminate in the activation of NF-κB, which plays an important regulatory role in the inflammatory immune responses (Kawai and Akira, 2007; El-Zayat et al., 2019). Dysregulation of TLRs and NF-κB activation disrupts immune homeostasis and leads to an exaggerated inflammatory response through excessive and sustained production of proinflammatory cytokines and chemokines (Gao et al., 2017; Liu et al., 2017).

Although the exact mechanisms underlying smoking-related immunopathology are not fully understood, there is ample evidence that smoking impairs both innate and adaptive immunity. It disrupts immunological homeostasis and plays a dual role in regulating immunity, both increasing pathological immune responses and weakening the normal defensive function of the immune system (Kianoush et al., 2017; Qiu et al., 2017). Cigarette smoking causes dysregulation of TLRs and NF-κB (Yang et al., 2006; Semlali et al., 2012), which plays an important role in the pathogenesis of various diseases, including atherosclerosis (Hovland et al., 2015; Salvador et al., 2016), type 2 diabetes (Jialal et al., 2014; Aly et al., 2020), hypertension (Lazaridis et al., 2021), and others more.

## 3 OxS, hyperinsulinemia and insulin resistance, subclinical inflammation and dysregulation of the SNS, the RAAS and the immune system: Interrelations

**FIGURE 1:**
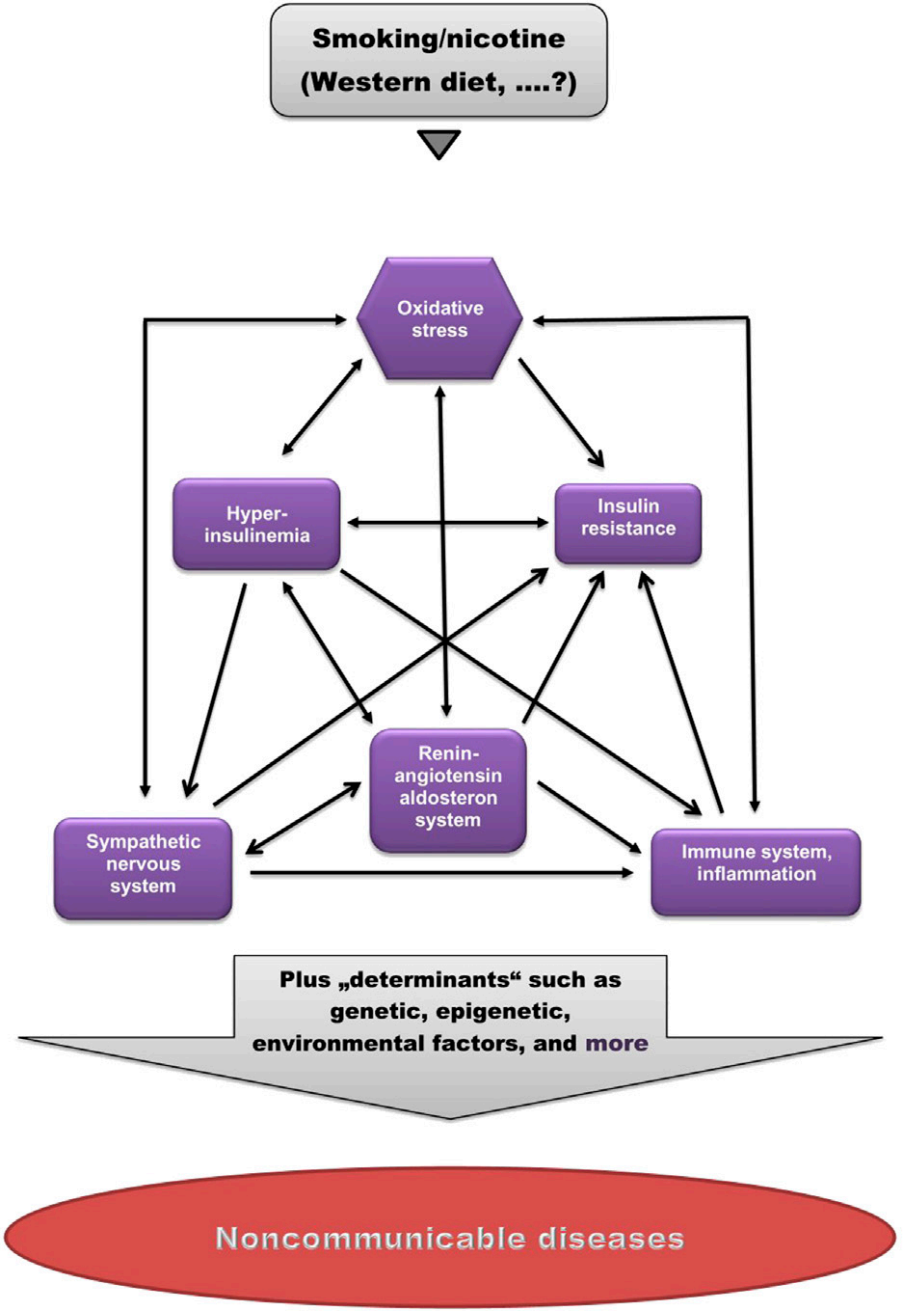
Pathogenetic model of (smoking-related) non-communicable diseases. Smoking/nicotine can cause non-physiological activation of each of these factors. Note the high degree of interrelations. (Modified from Kopp, 2019).

As shown in Figure 1, these factors are strongly interrelated, suggesting that dysregulation of one of them may cause imbalance in others.

### 3.1 OxS, immune system and inflammation

OxS plays a critical role in the development and maintenance of subclinical inflammation (Biswas, 2016; Forrester et al., 2018), while inflammation causes OxS, suggesting that inflammation and OxS are closely related pathophysiological events (Biswas, 2016).

### 3.2 OxS and RAAS

A very close relationship exists between OxS and RAAS overactivation. OxS and ANG II signaling mutually regulate each other by multiple mechanisms. Activation of the RAAS induces OxS, for instance in the vascular system or the kidney while OxS can activate the RAAS (Ramalingam et al., 2017). *In vitro* studies have shown that Ang II stimulates superoxide anion production in mesangial cells (Jaimes et al., 1998), whereas OxS was shown to be a direct stimulator of AT1R expression in an animal study (Banday and Lokhandwala, 2008), indicating a positive feedback loop.

### 3.3 OxS and insulin

Increased ROS production and OxS caused insulin resistance in vitro and in animal studies (Fazakerley et al., 2018). Exposure of ß-cells to mono-oleoyl glycerol induced insulin hypersecretion through the production of ROS, whereas ROS scavengers abolished the secretion (Saadeh et al., 2012). Chronic insulin treatment resulted in a significant increase in intracellular formation of superoxide anions, hydrogen peroxide, and hydroxyl radicals in 3T3-L1 fat cells, leading to the development of insulin resistance (Ge et al., 2008).

### 3.4 OxS and SNS

There is a positive feedback loop between sympathetic nerve activity and OxS (Middlekauff et al., 2014). OxS was shown to stimulate sympathetic nervous system activity in several experimental models of hypertension (Campese et al., 2005; Ye et al., 2006; Oliveira-Sales et al., 2008) while injection of antioxidants resulted in a decrease in sympathetic nervous system activity (Ye et al., 2006). Otherwise, NE increased superoxide production in human peripheral blood mononuclear cells *via* NOX activation and ROS production (Deo et al., 2013).

### 3.5 RAAS and immune system

ANG-II can cause dysregulations of the adaptive immune system through the stimulation of macrophages and other immune cells mediated by signalling through AT1R (Bernstein et al., 2018). In addition, Ang II can trigger activation of TLR4 *via* AT1R in various cell types, such as the kidney, vasculature, and central nervous system, resulting in an inflammatory immune response (Biancardi et al., 2017).

### 3.6 RAAS and insulin resistance

ANG II causes hyperinsulinemia and insulin resistance (Zhou et al., 2012; Mori et al., 2013). In an (*ex vivo*) hypertrophy model, hearts from ANG-II-treated mice exhibited decreased insulin sensitivity with markedly reduced glucose oxidation rates (Mori et al., 2013), whereas clinical trials in healthy subjects using the hyperinsulinemic euglycemic clamp technique showed that acute and chronic hyperinsulinemia increases RAAS activation (Rooney et al., 1991; Perlstein et al., 2007).

### 3.6 SNS and insulin resistance

Acute increases in plasma insulin (within the physiological range) stimulated a dose-dependent increase in SNS activity in normal subjects, as demonstrated by increased NE plasma concentrations and microneurographic studies (Rowe et al., 1981), whereas clinical trials in normal volunteer subjects showed that NE infusions cause insulin resistance (Lembo et al., 1994; Khoury and McGill, 2011), indicating the existence of a negative feedback loop (Kishi and Hirooka, 2013).

### 3.7 Hyperinsulinemia, insulin resistance and inflammation

*In vitro* studies showed that hyperinsulinemia causes insulin resistance *via* downregulation of insulin receptor phosphorylation (Catalano et al., 2014). Hyperinsulinemia has been shown to promote adipose tissue inflammation in mice, whereas a decrease in circulating insulin levels lead to a decrease in the expression of pro-inflammatory markers and macrophage content in adipose tissue (Pedersen et al., 2015). Hyperinsulinemia and hyperglycemia were (independently) shown to induce inflammatory responses in human chondrocytes *in vitro*, namely by activating NF-κB (Rufino et al., 2017).

Inflammation, particularly long-term chronic inflammation, may play important roles in development and progression of insulin resistance through multiple pathways (Chen et al., 2015; Pedersen et al., 2015; Wu and Ballantyne, 2020). Finally, insulin resistance generates compensatory hyperinsulinemia (Reaven and Tsao, 2003).

### 3.8 SNS and immune system

The SNS regulates many immune system functions (Nance and Sanders, 2007; Padro and Sanders, 2014) and is causally related to the development of chronic subclinical inflammation (Karakas et al., 2018).

### 3.9 SNS and RAAS

The SNS and the RAAS interact with each other in the form of a positive feedback loop: NE activates ANG II production by stimulating renin secretion, whereas circulating ANG II interacts with the SNS and potentiates NE release from sympathetic nerve terminals (Saxena, 1992; Grassi, 2001).

## 4 Non-communicable diseases caused by smoking: Dysregulation of the SNS, RAAS, and immune system, and disruption of physiological insulin and oxidant/antioxidant homeostasis as common underlying causative factors

### 4.1 Cardiovascular diseases

CVDs is a general term for a wide range of diseases, including diseases of the heart muscle and the vascular system that supplies the heart, brain, and other vital organs.

#### 4.1.1 Atherosclerosis

Atherosclerosis, the underlying cause of most CVDs, is the most important source of morbidity and mortality in the world (Barquera et al., 2015). Atherosclerosis is an inflammatory disease of the arterial wall, characterized by chronic inflammation and altered immune response. The pathogenesis of atherosclerosis involves at least three serious aspects: endothelial dysfunction, inflammation, and alterations in lipid metabolism. The early phase of atherosclerosis is associated with dyslipidemia and endothelial dysfunction. The endothelium is an active inner layer of the blood vessels and an important regulator of vascular tone by the production of NO. It also prevents leukocyte adhesion and platelet aggregation and maintains vascular health. Endothelial dysfunction is characterized by an imbalance between vasodilation and vasoconstriction, a deficiency of NO, platelet aggregation, thrombus formation, and increased vascular endothelial permeability. Vascular inflammation is involved in all stages of the atherosclerotic process, from lesion formation to plaque rupture and thrombus formation (Park and Park, 2015; Poznyak et al., 2020). Atherogenic dyslipidemia, defined as elevated triglyceride levels, high levels of small low-density lipoprotein cholesterol (LDL) and low levels of high-density lipoprotein cholesterol, also plays a pivotal role in CVD development (Borén and Williams, 2016). In a situation of increased endothelial permeability, high plasma LDL levels result in an increased rate of entry into the intima, and consequently, higher intimal LDL deposition. Deposition of cholesterol in the subendothelial area with subsequent sequestration and oxidation leads to activation of the innate and adaptive immune system and chronic vascular inflammation. Oxidized LDL, one of the major autoantigens in atherosclerosis, leads to the formation of foam cells and fatty streaks in the vessel wall, a hallmark of the onset of atherosclerosis (Boren et al., 2020; Poznyak et al., 2020). Various cell types, including macrophages, lymphocytes, endothelial cells, and smooth muscle cells, are involved in atherosclerotic lesion formation (Falk, 2006). Activation, proliferation and migration of vascular smooth muscle cells are crucial in both early and late stages of atherosclerosis. Vascular smooth muscle cells invade the early atherosclerotic lesion from the media and enlarge the lesions, but also form a protective fibrous cap (Chistiakov et al., 2015; Grootaert and Bennett, 2021). As the disease progresses, rupture or erosion of an atherosclerotic plaque, with subsequent thrombus formation and occlusion of the artery, may occur (Poznyak et al., 2020).

Vascular OxS (Poznyak et al., 2020), hyperinsulinemia (Arcaro et al., 2002), and dysregulation of the SNS (Grassi, 2001; Karakas et al., 2018), the systemic and local vascular RAAS (Pacurari et al., 2014), and the immune system (Hovland et al., 2015; Salvador et al., 2016) play key roles in the developmental process.

NOXs, an important source of cellular ROS, are involved in a variety of processes of atherogenesis. Numerous studies have shown that the upregulation of NOXs in vascular tissues and cell types such as endothelial cells, smooth muscle cells, fibroblasts, and others plays a key role in atherogenesis by promoting endothelial dysfunction and vascular inflammation. Of the 4 NOX isoforms expressed in human vascular tissue, NOX1 and especially NOX2 have been shown to promote atherogenicity (Kuntic et al., 2020; Poznyak et al., 2020). An *in vivo* study in NOX2 knockout mice has shown that knocking out NOX2 completely prevents the cardiovascular and cerebral side effects of e-cigarette smoking. In addition, *in vitro* studies using acrolein have replicated much of the NOX-2-associated vascular dysfunction caused by electronic cigarettes (Kuntic et al., 2020).

There is increasing evidence for an interaction between ROS-generating enzymes, such as mitochondrial oxidases, and NOXs as important sources of vascular ROS production (Poznyak et al., 2020). Also, ANG II-mediated OxS contributes to the initiation and maintenance of endothelial dysfunction, vascular inflammation, and vascular remodeling (Ekholm and Kahan, 2021; Poznyak et al., 2021). In addition, the RAAS stimulates accumulation of low-density lipoproteins, especially the oxidatively modified form, while (oxidized) lipid accumulation enhances the expression of RAAS components in blood vessels. This cross-talk between dyslipidemia and RAAS plays an important role in the atherosclerotic process (Singh and Mehta, 2003). As shown in a human clinical trial, even moderate hyperinsulinemia, which is comparable to fasting hyperinsulinemia in insulin resistance, can cause severe endothelial dysfunction in large conduit arteries (Arcaro et al., 2002). Insulin resistance may also alter systemic lipid metabolism and lead to the development of atherogenic dyslipidemia (Ormazabal et al., 2018). Persistent SNS overactivity induces functional and structural changes in various organs, like heart and blood vessels (Borchard, 2001).

Smoking is the most preventable cause of CVD and is involved in all steps of the above cascade (Roy et al., 2017). Exposure to cigarette smoke (Kim et al., 2014), its major constituent nicotine (Petsophonsakul et al., 2021), and electronic cigarettes (El-Mahdy et al., 2022) causes vascular OxS that is mediated, at least in part, by NOXs. Smoking-induced OxS decreases NO bioavailability, leading to endothelial dysfunction and vascular remodeling, the development and progression of vascular inflammation, and oxidation of lipoproteins. Further, *in vitro* cellular studies have shown that constituents of cigarette smoke lead to depletion of the cofactor tetrahydrobiopterin in vascular endothelial cells, which is essential for endothelial NO synthase function, resulting in uncoupling and dysfunction of endothelial NO synthase and endothelial dysfunction (Abdelghany et al., 2018). In addition, smoking-derived superoxide anions react preferentially with NO rather than with its endogenous scavenger superoxide dismutase, promoting the formation of peroxynitrite. Peroxynitrite causes uncoupling and dysfunction of endothelial NO synthase and endothelial dysfunction, as demonstrated *in vivo* studies in smokers and *in vitro* studies in bovine aortic endothelial cells (Peluffo et al., 2009). Finally, peroxynitrite inactivates prostacyclin synthase by activating NF-κB and increasing nitric oxide synthase expression in endothelial cells *in vitro*, which also leads to endothelial dysfunction (Cooke and Davidge, 2002). As shown in *in vitro* studies of human gingival epithelial cells (Semlali et al., 2012) and macrophages (Yang et al., 2006), smoking leads to dysregulation of TLRs and NF-κB, which contribute to atherogenesis by promoting OxS, inflammation, and endothelial dysfunction (Hovland et al., 2015; Lazaridis et al., 2021). Clinical and experimental data suggest that platelets and the coagulation system also play an important role in atherogenesis and atherothrombosis, and that exposure to cigarette smoke has prothrombotic effects by impairing the functions of endothelial cells, platelets, fibrinolytic factors, and coagulation factors (Barua and Ambrose, 2013; Gutowska et al., 2019). Nicotine-mediated impairment of RAAS homeostasis causes endothelial dysfunction, vascular inflammation, vascular remodeling and increased arterial stiffness (Oakes et al., 2018). Finally, smoking causes insulin resistance, hyperinsulinemia and atherogenic dyslipidemia (Facchini et al., 1992; Frati et al., 1996; Li et al., 2019; Lan et al., 2020). (*Limitation: see discussion*).

#### 4.1.2 Hypertension

Essential hypertension (EH), one of the most common chronic diseases, is a major risk factor for stroke, myocardial infarction and heart failure and the leading cause of morbidity and mortality worldwide (Mills et al., 2020). It is characterized by significant and persistent elevations in arterial pressure. The etiology of EH is complex and multifactorial and still not fully understood. Genetic (familial predisposition), environmental and behavioral factors (such as high salt intake, psychological stress, obesity, gut dysbiosis) are known hypertensinogenic factors (Carretero and Oparil, 2000; Harrison et al., 2021). Abnormalities of cardiac, vascular, and renal function are central to the pathophysiology of EH. The balance between cardiac output and peripheral vascular resistance is an important factor in maintaining normal blood pressure. In most patients with EH, there is an imbalance between cardiac output and peripheral vascular resistance in favor of increased peripheral resistance caused mainly by persistent smooth muscle constriction in the small arterioles with subsequent thickening of the wall and narrowing of the lumen. In adaptation to the increased peripheral resistance, hypertrophy of the cardiac musculature develops. The kidney plays a key role in the development of EH. This is reflected in the fact that “blood pressure goes with the kidney”, as cross-transplantation experiments and findings from kidney transplantation in humans have shown. On the one hand, kidney dysfunction leads to an increase in blood pressure, on the other hand, high blood pressure accelerates the loss of function of the kidney (Carretero and Oparil, 2000; Harrison et al., 2021).

Dysregulation of multiple homeostatic systems, with damage to the vascular system, heart, and kidneys, are involved in the developmental process (Oparil et al., 2018; Harrison et al., 2021). OxS (Poznyak et al., 2020; Harrison et al., 2021), insulin resistance (Mancusi et al., 2020), dysregulation of the systemic and local vascular RAAS (Manrique et al., 2009), the SNS (Tsioufis et al., 2011; Harrison et al., 2021) and the immune system (Harrison et al., 2021; Lazaridis et al., 2021) are key factors in the developmental process and cause endothelial dysfunction, vasoconstriction, vascular inflammation and vascular remodeling, hallmarks of EH (Oparil et al., 2018; Lazaridis et al., 2021). Impaired endothelium-dependent relaxation and structural changes in arteries and arterioles lead to stiffening of these vessels and an increase in peripheral vascular resistance and arterial pressure. Subsequently, damage to the heart and kidneys may occur, leading to dysfunction of these organs and further progression of the disease (Oparil et al., 2018; Harrison et al., 2021). OxS plays an important role in renal dysfunction and cardiovascular injury. Studies have shown that patients with essential hypertension have excessive ROS levels associated with reduced antioxidant capacity. NOXs of the vessels, heart, kidneys and immune system are a major source of ROS and OxS in EH (Touyz et al., 2020). There is increasing evidence that mitochondrial OxS is also invoved (Harrison et al., 2021). Fasting and postprandial insulin levels are higher in untreated patients with EH compared with normotensive controls, and there is a direct relationship between plasma insulin concentration and blood pressure. Insulin resistance and compensatory hyperinsulinemia contribute to EH through multiple mechanisms, including OxS, increased tissue ANG II and aldosterone activity, and increased activation of the SNS (Mancusi et al., 2020).

While the results of epidemiological studies examining the effects of smoking on the development of EH are controversial (reviewed in Samadian et al., 2016), there are several lines of evidence supporting a causal relationship between smoking and EH: an *in vivo* mouse model provided convincing evidence that chronic cigarette smoking causes hypertension and cardiac remodeling (Talukder et al., 2011). Exposure to cigarette smoke (Kim et al., 2014), its major constituent nicotine (Petsophonsakul, 20121), and electronic cigarettes (El-Mahdy et al., 2022) causes vascular OxS mediated at least in part by NOXs, leading to endothelial dysfunction, vascular remodeling, and vascular inflammation, key factors in the development process (Doonan et al., 2010; Golbidi et al., 2020). As shown in *in vitro* studies of human gingival epithelial cells (Semlali et al., 2012) and macrophages (Yang et al., 2006), smoking leads to dysregulation of TLRs and NF-κB. TLRs are expressed not only on immune cells, but also on non-immune cells such as vascular endothelial and smooth muscle cells, as well as on cells of the kidneys and nervous system, which are the three major target organs in hypertension. TLR activation on immune and vascular cells leads to activation of downstream signaling pathways that trigger an immune response with upregulation of proinflammatory cytokines and activation of NOXs, which contribute to the development of hypertension by promoting OxS, inflammation, endothelial dysfunction, smooth muscle cell migration and proliferation, and ultimately organ damage (Hovland et al., 2015; Lazaridis et al., 2021). Impairment of RAAS homeostasis by chronic smoking or nicotine exposure leads to endothelial dysfunction, vascular inflammation, vascular remodeling, and increased arterial stiffness (Oakes et al., 2018). Exposure to cigarette smoke (Middlekauff et al., 2014; Xiao et al., 2019) and e-cigarette smoke (Dimitriadis et al., 2022) acutely exerts a hypertensive effect, mainly through its powerful sympathetic excitatory effect. (*Limitation: see discussion*).

### 4.2 Cancer

Cancer is a complex, multifactorial disease involving genetics, environment and lifestyle. Lung cancer is the leading cause of cancer-related deaths worldwide (Sung et al., 2021). An estimated 90% of lung cancer deaths are due to tobacco smoking. In addition, there is sufficient evidence of a causal relationship between tobacco smoking and an increased risk of cancer of the upper digestive tract, esophagus, stomach, bladder, kidney, colon, prostate, pancreas, and acute myeloid leukemia. Data on an association between tobacco smoking and liver, cervical, brain, gallbladder, Hodgkin’s disease, non-Hodgkin’s lymphoma, and hematologic malignancies are inconsistent and require further investigation (Khani et al., 2018). There is a strong dose-response relationship between duration and intensity of cigarette smoking and many cancers (Inoue-Choi et al., 2018).

Tobacco smoke is a toxic mixture of thousands of chemicals, at least 70 of which are carcinogenic. The most important carcinogens in humans include nicotine, nitrosamines and polycyclic aromatic hydrocarbons. On the one hand, carcinogens such as tobacco-specific nitrosamines and polycyclic aromatic hydrocarbons trigger cancer by causing DNA mutations and/or DNA and protein adducts; on the other hand, nicotine and nitrosamines promote cancer progression through receptor-mediated effects, like activation of non-neuronal nicotinic acetylcholine receptors and β-adrenergic receptors. Upon receptor activation, a wide range of signal transduction pathways is activated, which promote cancer cell growth, angiogenesis, migration, and invasion, and inhibit apoptosis (Xue et al., 2014; Nooshinfar et al., 2017).

Metabolically activated tobacco smoke carcinogens directly cause mutations observed in tumor suppressor genes and oncogenes (Hecht, 2012). In addition to smoke-related carcinogens, OxS, hyperinsulinemia and insulin resistance, inflammation, the SNS and the RAAS were identified as important causal factors for cancer development and progression. OxS and inflammation play an essential role in tumorigenesis by promoting multiple oncogenic events, including cell proliferation, angiogenesis, migration, metabolic reprogramming, and evasion of apoptosis in cancer cells (Hayes et al., 2020; Vaidya et al., 2020). Inflammation predisposes to the development of cancer and plays an important role in all stages of tumor development; cancer cells, together with their surrounding stromal and inflammatory cells, form an inflammatory tumor microenvironment which contributes to proliferation, angiogenesis, metastasis and more (Greten and Grivennikov, 2019). A growing body of evidence suggests that dysregulation of TLRs plays a critical role in cancer development and progression by modulating the inflammatory microenvironment (Mokhtari et al., 2021). Elevated insulin levels have been associated with increased cancer risk and progression in epidemiological studies. Both insulin itself and insulin-like growth factors 1 and 2 are potent mitogens that play important roles in promoting cell proliferation, differentiation, metastasis, and inhibition of apoptosis (Gallagher and LeRoith, 2020; Yee et al., 2020). Insulin receptors are normally downregulated in response to hyperinsulinemia. In cancer cells, however, insulin receptors may be over-expressed 2–6 fold which further exposes them to growth stimulation by insulin (Milazzo et al., 1992). In addition, hyperinsulinemia leads to a decrease in sex hormone-binding globulin, which can increase the availability of free sex hormones and promote the development of sex hormone-dependent cancers such as breast, prostate, and endometrial cancers (Arcidiacono et al., 2012). The RAAS also plays a crucial role in cancer biology and affects development and maintenance of cancer directly and indirectly by remodeling the tumor microenvironment. It regulates almost all hallmarks of cancer including tumorigenesis, proliferation, angiogenesis, cell migration and metastasis (Pinter and Jain, 2017; Rasha et al., 2019). Finally, β-adrenergic signaling has been shown to promote several cellular processes that contribute to cancer initiation, progression, and metastasis, including proliferation, inflammation, angiogenesis, immune escape, epithelial-mesenchymal transition and invasion of the extracellular matrix (Mravec et al., 2020; Conceição et al., 2021). (*Limitation: see discussion*).

### 4.3 Type 2 diabetes

Diabetes is one of the most common chronic metabolic disorder caused by an interplay of genetic, environmental and lifestyle factors. It is characterized by defective glucose metabolism as a result of impaired pancreatic β-cell function and insulin resistance (Galicia-Garcia et al., 2020). Many epidemiological studies have shown that cigarette smoking is one of the most important modifiable risk factors for type 2 diabetes, and most studies showed a dose-response relationship between smoking and diabetes (Śliwińska-Mossoń and Milnerowicz, 2017). In addition, cigarette smoking worsens metabolic control in diabetic patients. In a cross-sectional clinical study, smokers had on average 15%–20% higher insulin requirements and higher serum triglyceride concentrations compared with non-smokers, increasing to 30% in heavy smokers (Madsbad et al., 1980).

Normal glucose homeostasis requires fine tuning of insulin secretion by pancreatic β-cells in response to changes in blood glucose levels. Type 2 diabetes is characterized by insulin resistance, impaired hepatic glucose homeostasis and dysregulated insulin secretion. Initially, insulin resistance is compensated by increased insulin secretion, but over time, ß-cell dysfunction and diabetes can develop (Galicia-Garcia et al., 2020). Mitochondrial OxS plays a critical role in the development of insulin resistance (Fazakerley et al., 2018) and insulin hypersecretion (Saadeh et al., 2012). It further can induce β-cell dysfunction due to the low antioxidant capacity of beta cells (Eguchi et al., 2021). *In vitro* studies in ß-cells/islets and *in vivo* animal studies showed that ANG II causes β-cell inflammation and β-cell dysfunction through induction of endoplasmic reticulum stress (Chan et al., 2017). NOX-induced vascular OxS is also associated with the development and progression of the major vascular complications of diabetes (Urner et al., 2020). In the Atherosclerosis Risk in Communities Study, which enrolled more than 8,000 non-diabetic middle-aged adults, SNS overactivity nearly doubled the risk of type 2 diabetes during an eight-year follow-up period (Carnethon et al., 2003). Smoking-related dysregulation of TLRs and NF-κB (Yang et al., 2006; Semlali et al., 2012) may play a role in the development of type 2 diabetes and its complications. TLRs, particularly TLR2 and TLR4, are involved in the development and progression of insulin resistance and diabetic complications such as diabetic nephropathy and vascular damage (Jialal et al., 2014; Aly et al., 2020). A clinical study in type 2 diabetic patients with or without renal insufficiency showed that the expression of TLR2 and TLR4 was higher in patients with renal insufficiency than in patients without renal insufficiency or in normal subjects and correlated positively with the degree of insulin resistance and negatively with the degree of insulin sensitivity (Aly et al., 2020). Nicotine also may be at least partially responsible for the development of diabetes. On the one hand, long-term use of nicotine gum is associated with insulin resistance and hyperinsulinemia (Eliasson et al., 1996). In addition, nicotine can affect insulin secretion *via* neuronal nicotinic acetylcholine receptors expressed on beta cells. Acute nicotine exposure at concentrations greater than 1 μmol/L inhibited high glucose-induced insulin release in isolated human islet cells *in vitro* (Yoshikawa et al., 2005). (*Limitation: see discussion*).

### 4.4 Osteoporosis

Osteoporosis is a chronic metabolic disease of the skeleton characterized by loss of bone mass and deterioration of bone quality, leading to increased susceptibility to fractures. Osteoblasts and osteoclasts are the main cells responsible for bone remodeling. Their activity is regulated by various factors, including the RANKL-RANK-OPG signaling pathway, estradiol, various cytokines and calciotropic hormones (Al-Bashaireh et al., 2018).

Cigarette smoking creates an imbalance in bone turnover, which leads to lower bone mass and decreased bone mineral density and makes bones prone to osteoporosis and fractures. Smoking was therefore classified as a risk factor for osteoporosis and fractures and included in the Fracture Risk Assessment Tool (Al-Bashaireh et al., 2018). The effects of tobacco smoke on bone health are complex, and several mechanisms are thought to play a role, including alterations in the metabolism of calciotropic hormones and intestinal calcium absorption, disruptions in the production and metabolism of sex hormones, alterations in the hormonal metabolism of the adrenal cortex and in the system of receptor activators of NF-κB and osteoprotegerin (Yoon et al., 2012; Al-Bashaireh et al., 2018). Chronic exposure to cigarette smoke caused enhanced osteoclast function by upregulating the RANKL/OPG ratio in an *in vitro* bone co-culture system, resulting in an osteoporotic microenvironment (Zhu et al., 2020). In postmenopausal women, bone mineral density was significantly lower in smokers than in non-smokers in a human clinical study. An additional animal study showed that administration of nicotine to wild-type mice decreased bone mass (Kiyota et al., 2020).

In a human cross-sectional study on 612 participants, smoking was associated with lower serum levels of vitamin D than non-smokers, with a dose-response pattern (Jiang et al., 2016). The exact mechanisms by which smoking affects vitamin D metabolism are still unclear. It is worth noting, however, that high insulin levels— common in smokers (Eliasson et al., 1994)—can lower vitamin D levels (De Pergola et al., 2013). Also, chronic overstimulation of the RAAS is thought to result in lower vitamin D levels (Ferder et al., 2013). Smoking cessation was associated with a significant increase in bone mineral density within a short period of time in a human clinical trial and in an animal study, as reflected by a significant decrease in serum levels of TRAP5b, a marker of bone resorption, and an increase in levels of osteocalcin and non-carboxylated osteocalcin (Kiyota et al., 2020).

In addition to the above factors, other factors play important roles in osteoporosis. OxS influences the bone remodeling process and causes an imbalance between osteoclast and osteoblast activity (Callaway and Jiang, 2015). Smoking-related OxS can increase bone resorption and contribute to lower bone mass (Kohler et al., 2021). Subclinical inflammation and SNS and RAAS imbalances are also associated with bone resorption and osteoporosis. Clinical and molecular evidence suggests that subclinical inflammation has a significant impact on bone turnover and the development of osteoporosis (Ginaldi et al., 2009). Chronic subclinical inflammation is an important trigger for osteoclast differentiation, which increases bone resorption (Kalyan et al., 2017). OxS and the formation of advanced glycation end products act as the link between inflammation and bone resorption (Pietschmann et al., 2016). There is ample evidence that the SNS is closely linked to bone remodeling. Increased SNS activity leads to bone loss through increased bone resorption and decreased bone formation mediated by β2-adrenergic influence on osteoblastic and osteoclastic cells (Farr et al., 2012; Kim et al., 2017). In a human clinical study of 23 women (10 premenopausal, 13 postmenopausal), sympathetic activity measured by microneurography was inversely related to bone volume and trabecular microstructure. Moreover, sympathetic activity in a subgroup of postmenopausal women correlated negatively with serum levels of procollagen type I N-peptide, a marker of bone formation in osteoporosis (Farr et al., 2012). Dysregulation of the RAAS has also been implicated in the development of osteoporosis. Ang II accelerates osteoporosis by activating osteoclasts and inhibiting osteoblast differentiation and bone formation (Chen, et al., 2017; Mo et al., 2020). (*Limitation: see discussion*).

### 4.5 Polycystic ovary syndrome

Polycystic ovary syndrome (PCOS) is one of the most common endocrine and metabolic disorders in premenopausal women. It can affect the endocrine, reproductive, metabolic, and psychological health of women beginning at puberty. The etiology of this syndrome remains largely unknown, but there is growing evidence that a genetic predisposition, as well as epigenetic and environmental factors, including diet and lifestyle, are involved in the developmental process. A recently published meta-analysis of Mendelian randomization studies has shown that a genetic predisposition to smoking (smoking initiation or lifetime smoking) is a causal risk factor for PCOS (Larsson and Burgess, 2022).

PCOS is associated with abnormal hormone production and metabolism, with elevated androgen levels and a high ratio of luteinizing hormone to follicle-stimulating hormone being among the fundamental alterations. Hyperandrogenemia is considered the main clinical hallmark of PCOS. The pathogenesis of PCOS is complex and not yet fully understood. Hyperinsulinemia and insulin resistance play a key role in the pathogenesis of PCOS. Insulin stimulates the theca cells of the ovary to produce excess testosterone (mainly attributed to a steroidogenic defect in theca cells) and suppresses the level of sex hormone-binding globulin, resulting in an increase in free testosterone (Sanchez-Garrido and Tena-Sempere, 2020; Rostamtabar et al., 2021).

Systemic and local ovarian SNS hyperactivity has been linked to the pathogenesis of PCOS, although the exact mechanism is not fully understood (Davis et al., 2019). Direct intraneural recordings of sympathetic nerve activity in the muscle vascular bed in women with PCOS showed that PCOS is associated with increased sympathetic nerve activity (Sverrisdottir et al., 2008). Increased sympathetic tone is associated with elevated androgen levels, anovulation, and menstrual irregularity and may play a role in the pathogenesis of the disease (Sverrisdottir et al., 2008; Li et al., 2014; Davis et al., 2019).

In recent years, a growing number of studies suggest that PCOS is a (pro)inflammatory disease and that chronic, low-grade inflammation is an important factor in ovarian dysfunction and the development of associated metabolic abnormalities. OxS plays an important role in chronic low-grade inflammation and is considered a potential triggering factor in the pathogenesis of PCOS. Markers of OxS and inflammation correlate strongly with circulating androgens (González, 2012; Rostamtabar et al., 2021). ROS play an important role in regulating ovulation and follicular dynamics. Increased ROS production may stimulate inflammatory signaling pathways, leading to ovarian cyst dysfunction and disruption of normal ovulation (Boots and Jungheim, 2015). CYP17, an important component of the androgen synthesis pathway, was upregulated in interstitial theca cells by proinflammatory stimuli and inhibited by resveratrol *in vitro* (Ortega et al., 2014). (*Limitation: see discussion*).

### 4.6 Chronic obstructive pulmonary disease

COPD is the third leading cause of death globally. COPD is characterized by chronic airway inflammation, emphysema and bronchiolar obstruction, with impaired lung function. Cigarette smoking is the major risk factor for COPD (Hikichi et al., 2019; Agusti et al., 2020). Exposure to tobacco and/or e-cigarette/nicotine vapor causes significant OxS and inflammation (Zuo et al., 2014). OxS (Boukhenouna et al., 2018; Barnes, 2022) and increased activation of the RAAS (Tan et al., 2018; Mei et al., 2020) are essential driving mechanisms in the pathogenesis of COPD, predominantly due to their inflammatory potential. There is growing evidence that COPD is associated with lung-specific and systemic immune dysfunction that triggers chronic inflammation and subsequent tissue destruction (Rovina et al., 2013; Bhat et al., 2015). Numerous recent data support an important role for TLRs in the initiation and development of the inflammatory process in COPD (Sidletskaya et al., 2020). Hyperinsulinemia and insulin resistance have also been implicated in the developmental process of COPD. In a human clinical trial, hyperinsulinemia and insulin resistance were demonstrated in COPD patients compared to healthy controls (Ruan et al., 2019). Hyperinsulinemia contributes to the inflammatory process and has adverse effects on the structure and function of the airways (Singh et al., 2016; Ruan et al., 2019). Experimental data show that insulin exerts a number of remodeling effects on airway smooth muscle cells, including the ability to increase proliferation of primary human airway smooth muscle cells, thereby affecting lung structure and function (Sagun et al., 2015). Furthermore, insulin can lower vitamin D levels (De Pergola et al., 2013), a factor that has been linked to the developmental process of COPD (Janssens et al., 2011). Finally, human clinical studies revealed that COPD is associated with increased sympathetic nervous system activity, which may contribute to progression and adverse outcome of the disease (Heindl et al., 2001; Andreas et al., 2014). (*Limitation: see discussion*).

### 4.7 Psoriasis

Psoriasis is a chronic, systemic immune-mediated disease with skin and joint manifestations. It is associated with hyperproliferation and dysfunctional differentiation of keratinocytes. Both genetic and environmental factors are involved in the developmental process. Smoking has been associated with the onset of psoriasis and has also been linked with the severity of the disease (Naldi, 2016; Zhou et al., 2020). A recently published review and meta-analysis of sixteen case-control studies found that heavy smokers and those who had smoked for a long time were particularly at increased risk for developing psoriasis (Zhou et al., 2020).

Insulin sensitivity has been reported to be significantly lower in patients with psoriasis compared to control subjects (Gyldenløve et al., 2015). In a large cohort study, including 21,789 postmenopausal women, insulin resistance was significantly associated with an increased risk of psoriasis during a cumulative 21-year follow-up (Chan et al., 2022). Several human clinical trials found a significant positive correlation of serum insulin levels and insulin resistance indices with psoriasis severity (Napolitano et al., 2015).

Also, overactivation of the local RAAS as a result of increased ACE expression (Silva et al., 2020), and smoking-related OxS and inflammation (Armstrong et al., 2011; Pleńkowska et al., 2020) were found to play important roles in the pathogenesis of the disease. There is growing evidence that TLRs participate in the pathogenesis of various inflammatory skin diseases, including psoriasis. Abnormal activation of TLRs leads to an exaggerated autoimmune response with increased production of cytokines such as IL17A and IL22, which are responsible for hyperproliferation and abnormal differentiation of keratinocytes, ultimately leading to the formation of psoriatic plaques (Sun et al., 2019). (*Limitation: see discussion*).

### 4.8 Multiple sclerosis

Multiple sclerosis (MS) is a chronic, potentially disabling disease of the SNS, characterized by inflammation, demyelination and axonal degeneration. Many genetic and environmental factors have been shown to contribute to its development, including cigarette smoking, Epstein-Barr virus infections, vitamin D deficiency, and obesity (Rosso and Chitnis, 2020).

OxS has been suggested to play a key role in the development of demyelination and axonal damage in both MS and its animal models (Ohl et al., 2016). In the acute phase OxS triggers inflammatory processes, in the chronic phase it maintains neurodegeneration. OxS was found to be associated with mitochondrial dysfunction, dysregulation of axonal bioenergetics and iron accumulation in the brain (Adamczyk and Adamczyk-Sowa, 2016). Recent research has shown that TLRs play a crucial role in the pathogenesis and progression of neuroimmune diseases by triggering an inflammatory response (Li et al., 2021). The expression of TLR3 and TLR4 was significantly increased in the center of MS lesions and around inflamed vessels in human postmortem brain tissues (Bsibsi et al., 2002). In addition, overactivation of the RAAS was found to play a pivotal role in the autoimmune inflammatory process. In a model of experimental autoimmune encephalomyelitis, quantitative RT-PCR analyses revealed upregulation of renin, angiotensin-converting enzyme, and AT1R in the inflamed spinal cord and immune system during autoimmune inflammation (Stegbauer et al., 2009). Insulin resistance and hyperinsulinemia are also common in patients with MS, as has been demonstrated in a human clinical trials (Penesova et al., 2015; Ruiz-Argüelles et al., 2018) and are thought to contribute to metabolic complications and overall impairment (*Limitation: see discussion*).

### 4.9 Crohn’s disease

Crohn’s disease is an idiopathic, chronic inflammatory process that can affect any part of the gastrointestinal tract, but often affects the colon and the terminal ileum. Cigarette smoking is a major risk factor for Crohn’s disease (Berkowitz et al., 2018; Papoutsopoulou et al., 2020). OxS, inflammation, hyperactivation of the immune system and upregulation of the RAAS are involved in the developmental process. Chronic inflammation and immune system hyperactivation are associated with OxS, and OxS is thought to play an important role in the development and maintenance of inflammation and abnormal immune responses (Alzoghaibi, 2013; Luceri et al., 2019). Recently, there has been increasing evidence that immune dysfunction, particularly TLR-mediated dysfunction of the innate immune system, plays a central role in the pathogenesis of Crohn’s disease. As has been shown, most members of the TLR family are involved in the progression of this disease (Lu et al., 2018). Activation of the local mucosal RAAS may promote development of Crohn’s disease and inflammation. In a transgenic mouse model that overproduces active renin, overactivation of the RAAS was shown to promote colitis by stimulating intestinal epithelial cell apoptosis and mucosal TH17 responses (Shi et al., 2016). Renin expression was enhanced in colon biopsies from patients with Crohn’s disease, and renin and ANG II levels in the colon were markedly increased in a TNBS-induced experimental colitis model (He et al., 2019). Insulin hypersecretion was found in patients with Crohn’s disease in a human clinical study by Bregenzer et al. (2006), likely caused by an upregulated enteropancreatic axis. (*Limitation: see discussion*).

### 4.10 Rheumatoid arthritis

Rheumatoid arthritis (RA) is a autoimmune disease that is characterized by chronic inflammation, synovial hyperplasia and cartilage and bone destruction in multiple joints and is influenced by both genetic and environmental factors (Torres et al., 2017).

Smoking is considered one of the best-known environmental risk factors for the development and severity of RA. A large proportion of patients have a history of smoking, and in addition, smokers are at increased risk for more severe rheumatoid arthritis. Smoking is associated with the pathogenesis of RA primarily through promotion of OxS, impairment of the immune response, and likely through epigenetic changes (Ishikawa, and Terao, 2020; Alemany-Cosme et al., 2021). Human clinical trials showed that patients with active disease have high levels of OxS, which translates into increased lipid peroxidation, protein oxidation, and DNA damage. Impairment of the body’s enzymatic and non-enzymatic antioxidant defense systems contributes to tissue damage and thus, chronicity of the disease (Mateen et al., 2016; Alemany-Cosme et al., 2021). It has been established that a dysfunctional TLR-mediated response is characteristic of RA patients and contributes to the development of a chronic inflammatory state (Arleevskaya et al., 2020). In addition, insulin resistance and dysregulation of the RAAS are associated with disease development. Insulin acts as a critical modulator of the inflammatory response by regulating intracellular and intercellular signaling pathways in immune cells, cartilage and synovial tissue. The increased prevalence of insulin resistance in patients with rheumatoid arthritis correlates with disease activity and disease-specific factors such as chronic systemic inflammation (Tripolino et al., 2021). Both classical (Moreira et al., 2021) and local RAAS activation (Cobankara et al., 2005) play important roles in the pathogenesis of rheumatoid arthritis. Ang II is considered a potent proinflammatory mediator, and overexpression of AT2R has been demonstrated *in vitro* in the inflamed synovial tissue of RA patients (Terenzi et al., 2017). *(Limitation: see discussion).*

### 4.11 Alzheimer’s disease

Alzheimer’s disease (AD) is a neurodegenerative disorder that affects tens of millions of people worldwide (Alzheimer’s Association, 2020). Epidemiological studies, meta-analyses, and case-control studies show that cigarette smoking is associated with a significantly increased risk of neurodegenerative diseases such as Alzheimer’s disease and dementia (Cataldo et al., 2010; Rusanen et al., 2011). AD is a multifactorial disorder, and the multiple mechanisms associated with the disease are not completely clear. It is driven by the production and deposition of amyloid β peptide and intracellular accumulation of neurofibrillary tangles of hyperphosphorylated τ protein (Cheignon et al., 2018; Abeysinghe et al., 2020).

Cigarette smoking was associated with risk biomarkers for Alzheimer’s disease characterized by excessive OxS, neuroinflammation, and elevated β-amyloid 42 levels in a case-control study in humans (Liu et al., 2020). Also, another human clinical study showed that exposure to cigarette smoke produces significant OxS in the central nervous system (Durazzo et al. (2016). OxS plays an important role in the developmental process of AD (Verdile et al., 2015; Misrani et al., 2021). On the one hand, the brain is particularly susceptible to oxidative damage due to its high oxygen consumption, high content of polyunsaturated fatty acids, and relatively high content of redox transition metal ions; on the other hand, the level of antioxidants in the brain is very low. Numerous research studies have shown that lipid peroxidation is greatly increased in AD. The accumulation of amyloid-β protein triggered by ROS leads to the degradation of the lysosomal membrane and eventually contributes to the death of neurons (Verdile et al., 2015; Misrani et al., 2021). Dysregulation of TLRs plays an important role in the development of AD, particularly in the early stages of the disease, by affecting synaptic plasticity, microglial activity, τ phosphorylation, and inflammatory responses (Momtazmanesh et al., 2020). The RAAS is involved in the development and progression of AD by increasing amyloid-β production, OxS and inflammation, and decreasing the release of acetylcholine (Gebre et al., 2018). Emerging evidence from human clinical trials and animal studies suggests that hyperinsulinemia and brain insulin resistance also are involved in the developmental process through multiple pathways, including a decreased clearance of amyloid-β-peptide and phosphorylation of τ protein, hallmarks of AD, and through effects on vasoreactivity, lipid metabolism, and inflammation (Fishel, et al., 2005; Verdile et al., 2015; Kellar and Craft, 2020). Finally, overactivation of the SNS has been implicated in the development of AD. NE is thought to be functionally largely opposed to the neuromodulators serotonin, dopamine, acetylcholine and melatonin, which are distributed throughout the brain and modulate many processes in the pathophysiology underlying AD (Fitzgerald, 2021). (*Limitation: see discussion*).

This list could be continued, but would go beyond the scope of this publication.

## 5 Smoking and microbiome

The intestinal microbiome plays an important role in human health and also in the development of disease due to its interactions with the immune system. Dysbiosis of the gut microbiome has been linked to several diseases, including asthma, COPD, Crohn’s disease, ulcerative colitis, CVD, obesity, rheumatoid arthritis, systemic lupus erythematosus, central nervous system diseases and cancer. A growing number of human clinical trials and animal studies have shown that smoking alters the composition of the gut microbiome. However, the underlying mechanisms of how smoking affects the microbiome are still largely unknown (Gui et al., 2021; Martinez et al., 2021).

## 6 Discussion

Cigarette smoking is the leading cause of preventable deaths worldwide. Cigarette smoking has been implicated in the pathogenesis of a host of chronic non-communicable diseases. The mechanisms by which cigarette smoking induces and promotes these diseases are still under debate. This review shows that smoking, like the Western diet (Kopp, 2019), causes a significant distortion of physiological balance, characterized by dysregulation of the SNS, the RAAS and the immune system, as well as disruption of physiological insulin and oxidant/antioxidant homeostasis, manifested as OxS and insulin resistance. As shown in Figure 1, all of these factors are strongly interrelated, suggesting that dysregulation of one of them may cause imbalance in others.

The evidence presented further shows that these factors play a key role in the development of a broad spectrum of smoking-related diseases, including CVDs, COPD, cancer, type 2 diabetes, Crohn’s disease, rheumatoid arthritis, psoriasis, PCOS, osteoporosis, MS and AD.

The dysregulation of the above factors may affect other physiological factors as well, which then also contribute to disease development. For example, overactivation of the RAAS (Singh and Mehta, 2003) and insulin resistance (Ormazabal et al., 2018) alter systemic lipid metabolism, leading to the development of atherogenic dyslipidemia. Chronic overstimulation of the RAAS (Ferder et al., 2013) and hyperinsulinemia (De Pergola et al., 2013; Cooper et al., 2020) were found to cause low vitamin D levels. Increasing epidemiological and laboratory diagnostic evidence suggests that vitamin D deficiency is associated with the onset and progression of numerous chronic non-communicable diseases (Wang et al., 2017). Hyperinsulinemia may increase the availability of free sex hormones, thereby promoting the development of sex hormone-dependent cancers (Arcidiacono et al., 2012).

Since not all smokers develop one or more of these diseases, it is proposed that this disruption of physiological balance represents a kind of pathogenetic “basic toolkit” for the potential development of a range of non-communicable diseases, and that the decision of whether and what disease will develop in an individual is determined by other, individual factors (“determinants”), such as the genome, epigenome, exposome, microbiome, and others (Figure 1).

Often, more than one chronic disease develops in an individual, which is referred to as comorbidity (Valderas et al., 2009). The pathophysiological pattern common to these diseases may provide an explanation for the often poorly understood links between non-communicable diseases and disease comorbidities, like for instance CVDs and type 2 diabetes (Einarson et al., 2018), CVD, COPD and cancer (Buddeke et al., 2019), CVDs and osteoporosis (Farhat and Cauley, 2008), CVDs and AD (Stampfer, 2006), COPD and osteoporosis (Sarkar et al., 2015), CVDs and psoriasis (Hu and Cheng-Che, 2017), psoriasis and AD (Kim et al., 2020), and psoriasis with CVDs, cancers, type 2 diabetes and Crohn’s disease (Takeshita et al., 2017), to name but a few.

A limitation of this work is that the evidence that the development of the diseases described is due to smoking-induced dysregulation of the above physiological factors is partly indirect. This is because there is a partial lack of studies describing the direct smoking-related influence of these factors on disease development. In these cases, the evidence relies on a combination of studies showing the dysregulatory effect of smoking and/or cigarette smoke constituents on the above factors and studies showing the pathogenetic effect of these factors on various non-communicative diseases.

## 7 Summary and conclusion

The aim of this work was to address the question of whether the pathogenesis of smoking-related and diet-related non-communicable diseases follows the same pattern. The evidence presented shows that both cause a significant distortion of physiological balance characterized by dysregulation of a group of important, strongly interrelated physiological factors that play key roles in the developmental process of many (most?) non-communicable diseases.

The proposed pathophysiological process offers new insights into the development of non-communicable diseases and may influence the direction of future research in both prevention and therapy.

## Abbreviations

ACE, angiotensin-converting enzyme; AD, Alzheimer’s disease; ANG, angiotensin; AT1R, ANG II type 1 receptor; COPD, chronic obstructive pulmonary disease; CVD, cardiovascular disease; EH, essential hypertension; LDL, low-density lipoprotein cholesterol; MS, multiple sclerosis; NE, norepinephrine; NF-κB, nuclear factor-κB; NO, nitric oxide; NOX, nicotinamide adenine dinucleotide phosphate oxidase; OPG, osteoprotegerin; OxS, oxidative stress; PCOS, polycystic ovary syndrome; RA, rheumatoid arthritis; RAAS, renin-angiotensin aldosterone system; RANKL, receptor activator of nuclear factor (NF)-kB-ligand; RNS, nitrogen species; ROS, reactive oxygen species; RT-PCR, reverse transcription polymerase chain reaction; SNS, sympathetic nervous system; TLR, toll-like receptor.
